# PD-1 blockade immunotherapy as a successful rescue treatment for disseminated adenovirus infection after allogeneic hematopoietic stem cell transplantation

**DOI:** 10.1186/s13045-024-01557-2

**Published:** 2024-05-20

**Authors:** Fei Zhou, Feng Du, Ziyan Wang, Mengxing Xue, Depei Wu, Suning Chen, Xuefeng He

**Affiliations:** 1 The First Affiliated Hospital of Soochow University, National clinical research center for hematologic diseases, Jiangsu Institute of Hematology, 188 Shizi Street, Suzhou, 215006 China; 2 Soochow Hopes Hematonosis Hospital, Suzhou, 215006 China

**Keywords:** Programmed cell death 1 inhibitor, Disseminated adenovirus disease, Hematopoietic stem cell transplantation

## Abstract

Disseminated adenovirus infection is a complication with a relatively high mortality rate among patients undergoing hematopoietic stem cell transplantation. The low efficacy and poor availability of current treatment options are of major concern. Programmed cell death 1 (PD-1) blockade has been used to treat several chronic viral infections. Herein, we report a case of disseminated adenovirus infection in the early posttransplant period. The patient was diagnosed with diffuse large B-cell lymphoma at first and underwent 8 cycles of chemotherapy, including rituximab. She was subsequently diagnosed with acute myeloid leukemia and received haploidentical transplantation. She was diagnosed with Epstein‒Barr virus (EBV)-positive posttransplant lymphoproliferative disorder (PTLD) 2 months after the transplant, and 3 doses of rituximab were administered. The patient was diagnosed with disseminated adenovirus infection with upper respiratory tract, gastrointestinal tract and blood involved at 3 months after transplantation. She was first treated with a reduction in immunosuppression, cidofovir and ribavirin. Then, the patient received salvage treatment with the PD-1 inhibitor sintilimab (200 mg) after achieving no response to conventional therapy. The adenovirus was cleared 3 weeks later, and concomitant EBV was also cleared. Although the patient developed graft-versus-host disease of the liver after the administration of the PD-1 inhibitor, she was cured with steroid-free therapy. Therefore, PD-1 blockade immunotherapy can be considered a promising treatment option for patients with disseminated adenovirus infection after transplantation, with fully weighing the hazards of infection and the side effects of this therapy.

To the Editor,

The reported prevalence rate of Human adenovirus (HAdV) infection after hematopoietic stem cell transplantation (HSCT) ranges from 5–21% [1]. Approximately 10–20% of patients with HAdV infection may develop disseminated adenovirus disease (dAdV), with mortality rates ranging from 20 to 80% [2]. DAdV infection is characterized by systemic symptoms with adenoviruses detected in two or more organs, or adenoviruses detected in one organ accompanied by high viral copy numbers in blood (> 10E4 per milliliter) [3]. Reducing immunosuppression and using antiviral drugs, including cidofovir, are still the main treatment methods. The prognosis of patients with dAdV remains poor even if they are treated with combined therapy [3–5]. Herein, we report a patient with dAdV who was successfully treated with a PD-1 inhibitor rapidly and effectively. This is the first report of PD-1 inhibitors being applied to treat dAdV patients after transplantation.

## Case presentation

**Fig. 1:**
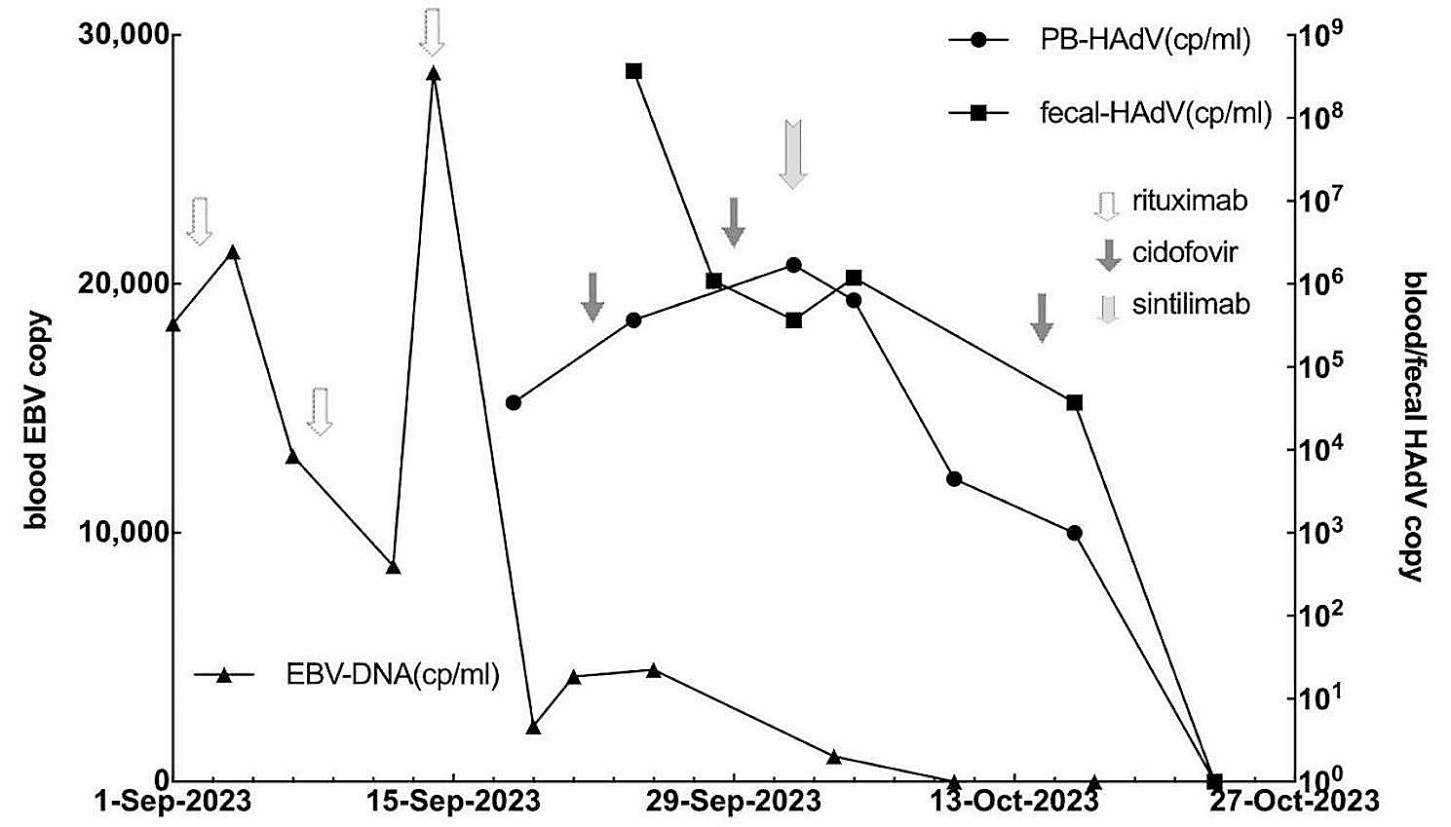
Treatment process and development trends of viral copy numbers in the patient

The patient was a 54-year-old female who was diagnosed with diffuse large B-cell lymphoma in March 2020. She underwent 8 cycles of chemotherapy, including rituximab, and reached complete remission. The patient was subsequently diagnosed with MLL-ELL-positive acute myeloid leukemia, presumed to be treatment-related, in October 2022 and underwent 3 cycles of chemotherapy. She reached morphological remission and received haploidentical transplantation from her son on June 14, 2023. Unfortunately, she was diagnosed with EBV-positive PTLD at 2 months posttransplant. Immunosuppressive agents were rapidly tapered, and 3 doses of rituximab (375 mg/m^2^) were administered. The number of EBV copies in the blood decreased from 4.1*10E5 to 4.5*10E3 per milliliter after treatment. Moreover, the patient got intermittent fever, nausea, vomiting, diarrhea, and cough, and HAdV was detected positive in blood, throat swabs and stool specimens, at 3.7*10E4 copies/ml (cp/ml) in blood and 4.0*10E8 cp/ml in stool at 3 months posttransplant. She was diagnosed with dAdV disease, with upper respiratory tract, gastrointestinal tract and peripheral blood involved. After administration of 2 doses of cidofovir (5 mg/kg per week), the patient’s clinical symptoms and inflammatory indicators worsened, and her HAdV copy number continued to increase to 1.7*10E6 cp/ml in blood. Since PD-1 inhibitors have been successfully used to treat several chronic viral infections [6–8], it is speculated that PD-1 inhibitors might be effective for HAdV clearance. Therefore, 200 mg of sintilimab (a recombinant human IgG4 monoclonal antibody against PD-1) was administered as a salvage treatment with the prior informed consent of the patient. The viral copy numbers in blood and stool both decreased gradually and finally became negative 3 weeks later (Fig. 1). The patient developed acute graft-versus-host disease (GVHD) grade III in the liver. She was treated with cyclosporine and 3 doses of basiliximab (20 mg, twice a week) and showed a complete response. Surprisingly, the patient also became negative for peripheral EBV soon after receiving sintilimab. Routine bone marrow exams at 4 months posttransplant showed molecular relapse of leukemia, with MLL-ELL being positive, so the patient was treated with 100 mg of sintilimab again. MLL-ELL turned negative 1 month later and no recurrence of GHVD occurred. At 9 months posttransplant, the patient was in good condition with no recurrence of leukemia, HAdV infection or GVHD.

## Discussion and conclusions

We treated the dAdV patient with sintilimab as a salvage therapy successfully and rapidly, though the additional efficacy of cidofovir cannot be completely ruled out. Concomitant EBV infection also seemed to respond to PD-1 blockade. Chen et al. reported that inhibitors targeting the PD-1 pathway could rescue T cells from an exhausted state and revive the immune response against EBV [9]. You et al. reported a successful case of sintilimab in the treatment of chronic active EBV infection after allogeneic HSCT [10], which, together with our case, suggests that PD-1 blockade has a curative effect on viral infections in HSCT setting.

The administration of PD-1 inhibitors in patients posttransplant has the risk of inducing GVHD, with one study showing that the incidence of GVHD can reach 55% [11]; hence the use of PD-1 inhibitors in the treatment of transplant recipients needs to be carefully managed. However, considering the relatively high mortality rate of dAdV infection, treatment with PD-1 inhibitors is still worth trying in patients with dAdV infection after HSCT.

## Data Availability

No datasets were generated or analysed during the current study.

## Abbreviations

PD-1: Programmed cell death 1
EBV: Epstein-Barr virus
PTLD: Posttransplant lymphoproliferative disorders
HAdV: Human adenovirus
HSCT: Hematopoietic stem cell transplantation
dAdV: Disseminated adenovirus disease
cp/ml: Copies per milliliter
GVHD: Graft-versus-host disease
